# EphA2-Dependent Internalization of *A. fumigatus* Conidia in A549 Lung Cells Is Modulated by DHN-Melanin

**DOI:** 10.3389/fmicb.2020.534118

**Published:** 2020-10-06

**Authors:** Esther M. Keizer, Han A. B. Wösten, Hans de Cock

**Affiliations:** Microbiology & Institute of Biomembranes, Department of Biology, Utrecht University, Utrecht, Netherlands

**Keywords:** *A. fumigatus*, EphA2, DHN-melanin, lung epithelial cells, internalization, aspergillosis

## Abstract

Dectin-1 and ephrin type-A receptor 2 (EphA2) receptors recognize β-glucan present in the fungal cell wall. Inhibition of Dectin-1 with the monoclonal 2a11 antibody was shown to reduce internalization of conidia of the human pathogen *Aspergillus fumigatus* into epithelial cells. In this study, we investigated the role of the EphA2 receptor present on A549 epithelial type II lung cells in the interaction with *A. fumigatus* conidia. We assessed whether EphA2 is involved in association and internalization of conidia by receptor inhibition by an antibody or by using the kinase inhibitor dasatinib. A 50% reduction of internalization of conidia was observed when this receptor was blocked with either the EphA2-specific monoclonal antibody or dasatinib, which was similar when Dectin-1 was inhibited with the 2a11 monoclonal antibody. Inhibition of both receptors reduced the internalization to 40%. EphA2 inhibition was also assessed in a hydrophobin deletion strain (Δ*rodA*) that exposes more β-glucan and a dihydroxynaphthalene (DHN)-melanin deletion strain (Δ*pksP*) that exposes more glucosamine and glycoproteins. The Δ*rodA* strain behaved similar to the wild-type strain with or without EphA2 inhibition. In contrast, the Δ*pksP* mutant showed an increase in association to the A549 cells and a decrease in internalization. Internalization was not further decreased by EphA2 inhibition. Taken together, the presence of DHN-melanin in the spore cell wall results in an EphA2-dependent internalization of conidia of *A. fumigatus* into A549 cells.

## Introduction

*Aspergillus fumigatus* is a saprotrophic fungus, which is able to colonize a large variety of dead organic material and living organisms (Krijgsheld et al., 2013). Colonies of this fungus produce asexual spores that are dispersed via the air. We inhale on average several hundred of these conidia per day (Mullins et al., 1984). Due to their small diameter of 2–3 μm (Brakhage and Langfelder, 2002), they can reach the deeper parts of the respiratory tract (Moore et al., 2011). The conidia can attach to lung epithelial cells, after which they can be internalized (Wasylnka and Moore, 2002). These characteristics contribute to the virulence of *A. fumigatus*, making it an opportunistic pathogen that can cause severe invasive infections in especially immunocompromised patients (Kosmidis and Denning, 2015).

The interaction of conidia and lung epithelial cells differs between fungal species and even strains. For example, no difference was observed between adherence of conidia of *Aspergillus niger* and *A. fumigatus* to A549 type II lung epithelial cells. In contrast, *A. fumigatus* conidia were internalized more efficiently, while germination within a 12 h period was also much less compared to *A. niger* (Escobar et al., 2016). Transcriptome analysis revealed that the immune response of the lung cells differs upon interaction with these two aspergilli. In contrast to *A. niger*, *A. fumigatus* downregulates a set of genes involved in the immune response. On the other hand, both aspergilli upregulate IL-8, being dependent on the MOI. A higher MOI results in increased IL-8 expression for both *A. fumigatus* and *A. niger* (Escobar et al., 2018).

One of the receptors of epithelial cells that are involved in the internalization of conidia and initiation of the immune response is Dectin-1. This is a c-type lectin receptor that recognizes the cell wall component β-1-3 glucan (Brown and Gordon, 2001). It is essential for the initiation of the immune response by producing inflammatory molecules (Brown et al., 2002). Together with Toll-like receptor 2 (TLR2), Dectin-1 is involved in the production of ROS and mediation of the inflammatory response of macrophages (Gantner et al., 2003). Inhibition of this receptor reduces internalization of *A. fumigatus* conidia in lung epithelial cells (Han et al., 2011). In the case of *Candida albicans*, it has been shown that the EphA2 is activated by β-glucan. This activation initiates the immune response and together with the EGFR EphA2 is involved in the endocytosis of *C. albicans* by the host cell (Swidergall et al., 2018). In addition, EphA2 is required for the antifungal activity of neutrophils and the control of fungal proliferation during *C. albicans* infection (Swidergall et al., 2019). In the case of *Cryptoccocus neoformans*, which can cause cryptococcal meningitis, the EphA2 receptor is important for the transport of the fungus across the blood–brain barrier (Aaron et al., 2018).

Dormant conidia of *A. fumigatus* are covered with a hydrophobic rodlet layer consisting of the rodlet proteins RodA and RodB, and a green pigment layer of 1,8-DHN-melanin (Latgé and Beauvais, 2014). Upon induction of germination, conidia start to swell and the rodlet and melanin layers fragment. As a result, conidia increasingly expose hydrophilic polysaccharide and glycoprotein patches, becoming completely hydrophilic at the end of the breakdown process (Dague et al., 2008). The rodlet and melanin layers thus cover immunogenic cell wall components like chitin, β-glucan, and glycoproteins, masking the dormant conidia for immune recognition (Latgé and Beauvais, 2014). Removal of the rodlet (Δ*rodA*) and/or the DHN-melanin layer (Δ*pksP*) leads to a rearrangement of the conidial cell wall. Conidia of the Δ*rodA* strain are more hydrophilic and expose more β-glucan (Carrion et al., 2013), while conidia of the Δ*pksP* strain expose more ConA reactive glycoproteins and glucosamine-containing components and chitin (Bayry et al., 2014; Valsecchi et al., 2019). Conidia of the double deletion strain (Δ*rodA*Δ*pksP*) expose more chitin but not more β-glucan (Bayry et al., 2014; Valsecchi et al., 2019). Inactivation of the melanin synthesis genes *ayg1* or *arp2* also results in reorganization of the conidial surface, similar to that of Δ*pksP*, which is explained by the absence of the early melanin intermediate scytalone. These cell wall rearrangements result in an increased immune response (Tsai et al., 1998; Bruns et al., 2010; Bayry et al., 2014).

In this study, we show that inhibition of EphA2 results in reduced internalization of dormant, swollen, or heat-killed conidia of *A. fumigatus*, but does not affect association of these conidia to the A549 lung epithelial cells. Notably, in contrast to the Δ*rodA* strain association of the Δ*pksP* strain to A549 cells was strongly increased, whereas the internalization decreased. No further decrease was observed upon EphA2 inhibition in the case of the Δ*pksP* strain. Dual inhibition of the EphA2 and Dectin-1 receptor showed a stronger reduction of internalization of Af293 and CEA10 strain when compared to the single inhibitors but internalization was not completely blocked. Taken together, DHN-melanin modulates the conidial internalization into A549 cells to an EphA2-dependent mechanism.

## Materials and Methods

### Strains and Growth Conditions

Strains used in this study (Table 1) were grown for 3 days on PDA (Difco) at 37°C. Conidia were harvested with 0.85% (w/v) NaCl and filtered through three layers of miracloth (Merck Millipore) to remove remnants of mycelium and hyphae. Conidia were counted using a Bürker Türk counting chamber.

**TABLE 1:** Strains used in this study.

Strain	Description	References
*A. fumigatus*		
Af293.1	pRG3AMA1-RFP	Leal et al., 2010
CEA10		Girardin et al., 1993
CEA10Δ*KU80*	pyrG^AF::Δ*ku80* in CEA10	da Silva Ferreira et al., 2006
CEA10Δ*pksP*	Δ*pksP*::hph in CEA10Δ*KU80*	This study
CEA10*pksP*C	*pksP*^+^ derivative of CEA10Δ*pksP*	This study
CEA10Δ*rodA*	Δ*rodA*::hph in CEA10Δ*KU80*	This study
*A. niger*		
AV112d.7	*PglaA*:dTomato	Vinck et al., 2011

### Transformation *A. fumigatus*

Knock-out vectors for *rodA* and *pksP* were created by amplifying their left flanks using primer pairs *rodA*LF*BamHI*FW/*rodA*LF*XbaI*Rev and *pksP*LF*BamHI*FW/*pksP*LF*XbaI*Rev, respectively (Table 2). Similarly, the right flanks were amplified using primer pairs *rodA*RF*XbaI*FW/*rodA*RF*PstI*Rev and *pksP*RF*XbaI*FW/*pksP*RF*SacI*Rev. Both flanks were ligated in *Bam*HI*/Pst*I and *Bam*HI*/Sac*I linearized pUC20 for r*odA* and *pksP*, respectively, using T4 polymerase according to the manufacturer’s protocol (Thermofisher Scientific). In the next step, a 3031 bp *Xba*I fragment containing the hygromycin resistant cassette (Punt et al., 1987) was ligated in between the left and the right flanks in the pUC20 derivatives after digestion with *Xba*I.

**TABLE 2:** Primers used in this study.

Name	Sequence
*rodA* LF *Bam*HI FW	5’ TAGGATCCCACGAGCCTGGCTAAAG 3’
*rodA* LF *Xba*I Rev	5’ GATCTAGAACAGCAGCGCTCAAAG 3’
*rodA* RF *Xba*I FW	5’ CCTCTAGACTACTCGTCGCTTCTG 3’
*rodA* RF *Pst*I Rev	5’ ATCTGCAGTTGTCGGCCTGGTTTG 3’
*pksP* LF *Bam*HI FW	5’ TAGGATCCGCACGGCCAACGTAG 3’
*pksP* LF *Xba*I Rev	5’ AGTCTAGATGGCGAGTGGTTTGC 3’
*pksP* RF *Xba*I FW	5’ ATTCTAGACCACGGCCATGAGTTCC 3’
*pksP* RF *Sac*I Rev	5’ CAGAGCTCAGCGCAGGATGACAGAC 3’
upstream *rodA* LF FW	5’ GTACGCATCTACGTGCTCCA 3’
hyg *rodA* Rev	5’ GTCCAAGCAGCAAAGAGTG 3’
upstream *pksP* LF FW	5’ GGAGATAGGTGCAGGTGTTC 3’
hyg *pksP* Rev	5’ GCCGTGGTTGGCTTGTATG 3’
*pksP* FW	5’ TGGCATTTGGGATAAGCACG 3’
*pksP* Rev	5’ GCAGGGCATGGCATTCTTAA 3’
*pksP* check FW	5’ CGACTCGATTGCATTGCTCA 3’
*pksP* check Rev	5’ CTGCTGTGCCAATTCATCGA 3’
plasmid FW	5’ TTATCTTTGCGAACCCAGGG 3’
plasmid Rev	5’ CAACCCTAGTACGCCCTTCA 3’

*Restriction sites within primers are underlined.*

*Aspergillus fumigatus* CEA10Δ*ku80* conidia were transformed using the PEG-mediated transformation protocol (Meyer et al., 2010). In short, conidia were grown overnight in complete medium consisting of minimal medium (MM; 6 g L^–1^ NaNO_3_, 1.5 g L^–1^ KH_2_PO_4_, 0.5 g L^–1^ KCl, 0.5 g L^–1^ MgSO_4_⋅7H_2_O, 0.2 mL L^–1^ Vishniac, pH 6.0) supplemented with 2 g L^–1^ tryptone, 1 g L^–1^ casamino acids, 1 g L^–1^ yeast extract, and 0.5 g L^–1^ yeast ribonucleic acids. Mycelium was isolated by filtering over a double layer of miracloth and incubated at 37°C and 80 r/min for a maximum of 60 min in lyzing enzymes from *Trichoderma harzianum* (Sigma Aldrich) in OM (0.01 M Pb buffer pH 5.8, 2.45 M MgSO_4_⋅7H_2_O). Protoplasts were filtered through a double layer of miracloth, collected by centrifugation for 10 min at 1120 *g*, and incubated for 5 min with PEG-6000 (Acros Organics) and a *Sma*I or *EcorV* linearized plasmid for the *pksP* and r*odA* deletion plasmids, respectively. The protoplasts were regenerated on MMS-agar [MM + 0.95M sucrose, 25 mg mL^–1^ caffeine (Sigma Aldrich), 100 μg mL^–1^ hygromycin (Sigma Aldrich), and 1.2% bacteriological agar (Scharlau)]. After 4–5 days, transformants were transferred to fresh MM plates with 50 μg mL^–1^ hygromycin. Genomic DNA was isolated from mycelium using phenol/chloroform, which was used to confirm deletion of the genes. To this end, primer pairs *upstreamrodA*LFFW/*h*yg*rodA*Rev and *upstreampksP*LFFW/*h*yg*pksP*Rev (Table 2) were used for PCR for the *rodA* and *pksP* deletion strains, respectively.

For complementation of strain CEA10Δ*pksP*, the *pksP* gene was amplified using primers *pksP*FW and *pksP*Rev (Table 2) and directly used for transformation. To distinguish transformants with a wild-type phenotype from wild-type contamination, plasmid pGDPGFP containing the reporter gene *GFP* (Lagopodi et al., 2002) was co-transformed enabling selection of fluorescent strains. Integration of the gene was checked with primers *pksP*checkFW and *pksP*checkRev, while presence of pGDPGFP was checked using primers plasmidFW and plasmidRev (Table 2 and Supplementary Figure 1).

### Cell Culture and Fungal Infections

The human lung carcinoma epithelial cell line A549 (ATCC, CCL-185) was maintained by serial passage in DMEM (Ref. code: 11995-065, Gibco) with 10% FBS (Gibco). Fungal infection experiments were done as described (Escobar et al., 2016). Briefly, cells were seeded at a concentration of 2.10^5^ cells mL^–1^ and cultured at 37°C and 5% CO_2_ until a confluent monolayer was formed consisting of 2.10^6^ cells mL^–1^. Cells in 12- or 48-well plates (Corning^®^, Costar^®^) were challenged with 2.10^5^, 2.10^6^, or 2.10^7^ conidia mL^–1^ DMEM, 10% FBS resulting in a MOI of 0.1, 1, or 10, respectively. Cells were cultured in 48-well plates containing 8 mm glass coverslips (ThermoFisher Scientific) for internalization and association experiments (see below).

### Internalization and Association of Conidia

Internalization and association experiments were performed as described (Escobar et al., 2016). In short, A549 cells were grown on 8 mm glass coverslips (ThermoFisher Scientific) until a confluent layer had been formed. A549 cells were incubated with dormant conidia, dormant heat-killed conidia, or swollen conidia. Spores were killed at 90°C for 20 min (Supplementary Figure 2), while swollen conidia were obtained by a 2 h incubation at 37°C in DMEM + 10% FBS. Dormant, swollen, or heat killed conidia that did not express a plasmid containing a fluorescent gene, such as *RFP* in Af293.1, were stained with 16–20 μg mL^–1^ *Aspergillus* FITC labeled antibody (ThermoFisher Scientific) for 60 min at room temperature before addition to the A549 cells. Unbound antibody was not removed by washing, to avoid loss of conidia during pelleting. Conidia were added to A549 cells at a MOI of 1 and incubated for 2 h. Unbound conidia were removed by washing three times with DMEM + 10% FBS after 2 h of incubation, after which incubation continued for 2 more hours. Conidia adhering to the A549 cells were visualized with 1% calcofluor white (CFW; Sigma Aldrich) in DMEM + 10% FBS. To this end, the dye was added for 10 min at 37°C followed by one washing step with DMEM + 10% FBS. Cells and conidia were fixed with 4% paraformaldehyde (PFA) (VWR international) for 5 min at 4°C and 20 min at room temperature. Background fluorescence of PFA was quenched with 20 mM NH_4_Cl (Acros Organics) for 20 min at room temperature. A549 cells were visualized with 1 μg mL^–1^ Hoechst (BD Biosciences). Coverslips were mounted onto glass slides using FluorSave^TM^ (Merck Millipore) and dried overnight followed by confocal analysis. To determine the number of conidia associated to the A549 epithelial cells (number of conidia per A549 cell), 10 fields at the coverslip were randomly chosen for imaging. Internalization was determined by analyzing z-stacks made at 10 randomly chosen sites at the coverslip. Conidia that were red or green were counted as internalized. On the other hand, conidia that were also stained with CFW were counted as adhering non-internalized spores, as CFW cannot penetrate into the A549 cells. Internalization values were expressed as the percentage of total conidia that associated with the cells. Experiments were done using biological triplicates. At least 100 conidia were counted per strain in each experiment.

### EphA2 and Dectin-1 Receptor Inhibition

EphA2 receptor activity was blocked by adding 2.5 μM dasatinib (Cell Signaling Technology) (Swidergall et al., 2018) or a 50 times dilution of the EphA2 antibody (#6997, Cell Signaling) 1 h prior to infection to the A549 cells. The Dectin-1 receptor was blocked by adding 20 μg mL^–1^ 2a11 antibody (ab82888, Abcam) for 1 h and washed three times with DMEM + 10% FBS before addition of the conidia (Brown et al., 2002).

### Surface Exposure of β-1-3 Glucan

Conidia were swollen for 2 h in DMEM + 10% FBS at 37°C before fixation with 4% PFA. Swelling of conidia was confirmed by staining with an 1,3 β-glucan antibody (2G8, ab233743 Abcam). PFA background fluorescence was quenched with 20 mM NH_4_Cl. Conidia were blocked with 0.3% BSA (Sigma) for 60 min. The 2G8 antibody was diluted 100-fold and incubated with conidia for 120 min. After washing once with PBS, the conidia were incubated with a 1000-fold dilution of the secondary antibody (Goat Anti-Mouse, Alexa Fluor^®^ 488, ab150113 Abcam) for 45 min. Conidia were mounted on a glass slide with FluorSave and dried overnight.

### Confocal Microscopy

Confocal images were acquired with a Zeiss LSM 700 microscope using the Plan-Apochromat 63 × 1.40 oil DIC (WD = 0.19) objective. Images were taken using the 405, 488, and 555 nm laser lines. Fluorescence emission of CFW and Hoechst was detected using the 400–490 nm spectral band. Red fluorescence emission of mRFP and dTomato was detected with the 560–700 nm spectral band and FITC fluorescence was detected with the 490–555 nm spectral band. Images were analyzed and processed with the Fiji image processing package of ImageJ^1^.

### Quantification of IL-8 Secretion

Confluent layers of A549 cells in 12 wells plates (Corning^®^, Costar^®^) were challenged with conidia for 2 h, after which unbound conidia were removed by washing three times with pre-warmed DMEM + 10% FBS. After washing, exposure was continued for 2 or 10 h. The culture medium was added to 96-well IL-8 ELISA plates (ThermoFisher Scientific) according to the manufacturer’s instructions. Experiments were done using biological triplicates.

### A549 Cell Damage

Confluent layers of A549 cells in 24-well plates (Corning^®^, Costar^®^) were challenged with conidia for 2 h, after which unbound conidia were removed by washing three times with pre-warmed DMEM. Exposure of the A549 lung cells to the conidia was continued for another 2 or 10 h. Cell damage after both periods of challenging (4 and 12 h in total) was measured by LDH released into the medium. The medium was added to a transparent 96-well plate (Corning^®^, Costar^®^) and LDH activity was measured using an LDH activity kit (Sigma Aldrich) according to manufacturer’s instructions. A549 cells not challenged with conidia served as control. Experiments were done using biological triplicates and technical duplicates.

### Statistical Analysis

Differences in association, IL-8 release, and LDH activity were analyzed using a one-way ANOVA with a *p*-value ≤ 0.05. A Tukey test with a Bonferroni correction for multiple testing was used as a *post hoc* test. For the analysis of the internalization of conidia, values were scored as in or out and treated as binary data. Differences were analyzed using a Pearson chi-square test with *p*-values ≤ 0.05 considered significant. Separate *t*-tests with a Bonferroni correction for multiple testing were used as a *post hoc* test.

## Results

### The Role of EphA2 in Conidial Internalization and Association

**FIGURE 1:**
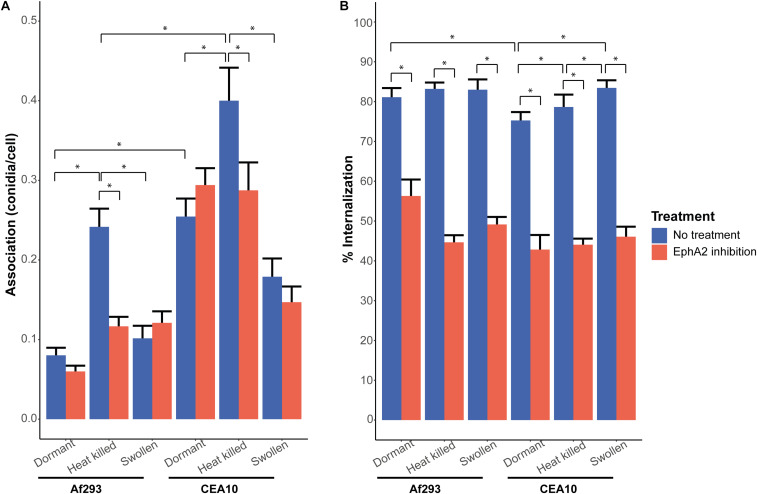
Internalization and association of *A. fumigatus* conidia of strains Af293 (expressing the *RFP* gene) and CEA10 WT (stained with the *Aspergillus*-FITC antibody) after 4 h of infection, with (red bar) or without (blue bar) inhibition of the EphA2 receptor by the EphA2 antibody. **(A)** Association of dormant, heat killed, or swollen Af293 and CEA10 conidia. **(B)** Internalization of dormant, heat killed, or swollen Af293 and CEA10 conidia. Bars represent the average of three separate experiments consisting of 10 pictures per condition/strain with the standard error. *Significant difference.

The EphA2 receptor of A549 epithelial lung cells was inhibited with a specific EphA2 antibody to assess the role of this receptor in association and internalization of conidia. To this end, the clinical *A. fumigatus* Af293 and CEA10 wild-type strains were used. Conidia were incubated with a confluent layer of A549 cells at a MOI of 1. The CEA10 and Af293 conidia associated to the A549 cells with efficiencies of 0.25 and 0.08 spores per cell within 4 h of incubation, respectively (Figure 1A). Inhibition of the EphA2 receptor did not result in a significant reduction of conidial association of both strains. Inhibition of the EphA2 receptor did reduce the internalization of conidia in A549 cells from 81 to 56% and 75 to 42% for Af293 and CEA10, respectively (Figure 1B). Thus, the efficiency of internalization of the two *A. fumigatus* strains into A549 epithelial lung cells differs and this process proceeds, at least in part, via the EphA2 receptor. The EphA2 receptor could also be inhibited with the broad range kinase inhibitor dasatinib. This inhibition resulted in a similar reduction of internalization of conidia from 93 to 51% and 73 to 47% for Af293 and CEA10, respectively (Supplementary Figure 3B). Association and internalization were assessed for the non-pathogenic *A. niger* N402 strain. Inhibition of EphA2 by dasatinib did not affect association to A549 cells but a similar decrease of internalization of conidia was observed as with the *A. fumigatus* strains (i.e., from 72 to 31%) (Supplementary Figure 4).

We next investigated if association and internalization of heat-killed and swollen conidia were affected by EphA2. The EphA2 receptor is activated upon recognition of β-glucan that is present in the fungal cell wall (Swidergall et al., 2018). We hypothesized that internalization of swollen conidia is more effective since β-glucan is less shielded by the rodlet and melanin layers when compared to dormant spores. It was observed that a 2 h incubation in culture medium resulted in increased exposure of β-glucan on the conidial surface of both strains (Supplementary Figure 5). Dormant conidia of CEA10 did expose more β-glucan as compared to Af293. In contrast, heat-killed conidia of both strains did not expose increased amounts of β-glucan at their surface when compared to the dormant spores. In fact, heat killing resulted in reduced detection of β-glucan in CEA10 (Supplementary Figure 5). Remarkably, internalization of heat-killed or swollen conidia of Af293 did not change significantly (Figure 1B) and thus does not correspond with β-glucan exposure at the surface of the swollen conidia (Supplementary Figure 5). In contrast, internalization of swollen and heat killed conidia of CEA10 increased with 8 and 4%, respectively (Figure 1B). Inhibition of the EphA2 receptor with a specific EphA2 antibody resulted in a reduction of internalization of swollen and heat-killed conidia from 83 to 49% and from 83 to 45%, respectively, for Af293 (Figure 1B) and a reduction from 83 to 46% and 79 to 44%, respectively, for CEA10 (Figure 1B). Internalization of swollen and heat-killed conidia upon inhibition of the EphA2 receptor by dasatinib resulted in a similar decrease in internalization (Supplementary Figure 3B). These results show that the increased exposure of β-glucan in swollen conidia does not increase the efficiency of internalization of conidia as compared to dormant conidia and that conidia do not need to be alive for efficient internalization.

Association of heat-killed conidia of Af293 was increased from 0.08 to 0.24 conidia per cell when compared to dormant conidia (Figure 1A), while association of swollen conidia of Af293 was similar to dormant conidia. This was not observed with conidia of CEA10. The association of heat-killed conidia increased from 0.25 to 0.4, while the association of swollen conidia decreased from 0.25 to 0.18 (Figure 1A). These results suggest that the swollen conidia of Af293 and CEA10 WT interact differently with the A549 epithelial lung cells. Association of heat-killed conidia of *A. niger* was reduced irrespective of the absence or presence of EphA2 (Supplementary Figure 4A), suggesting that conidia of *A. niger* N402 and *A. fumigatus* use different mechanisms for association to the A549 lung epithelial cells.

### Effect of DHN-Melanin and RodA Deletion on Conidial Internalization and Association

**FIGURE 2:**
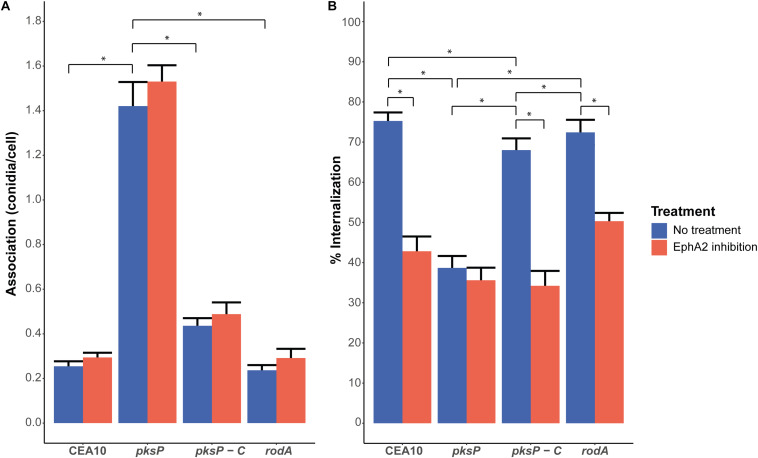
Association **(A)** and internalization **(B)** of conidia of the CEA10 WT strain, the Δ*pksP* (lacking DHN-melanin) and Δ*rodA* (lacking the hydrophobin RodA) deletion strains, and the complemented Δ*pksP* strain after 4 h of infection with (red bar) or without (blue bar) inhibition of the EphA2 receptor by an antibody. All conidia are stained with the *Aspergillus*-FITC antibody. Bars represent the average of three separate experiments consisting of 10 pictures per condition/strain with the standard error. *Significant difference.

Inactivation of the rodlet gene *rodA* results in increased exposure of β-glucan (Carrion et al., 2013) (Supplementary Figure 5), whereas deletion of the *pksP* gene results in increased glycoprotein and chitin exposure (Bayry et al., 2014; Valsecchi et al., 2019). The association of the conidia to the A549 lung cells was not significantly affected by the absence of the RodA protein (Figure 2A). Interestingly, a fivefold increase in association was observed in the case of Δ*pksP* conidia, whereas the *pksP* complementation strain had a similar association as the CEA10 wild-type strain (Figure 2A). Association of conidia of the Δ*rodA* and Δ*pksP* strains as well as the *pksP* complementation strain did not change when the EphA2 receptor was inhibited by the EphA2 antibody but did increase when the EphA2 receptor was inhibited by dasatinib (Figure 2A and Supplementary Figure 6A), contrasting their cognate wild-type strain. Deletion of *rodA* in CEA10 did not alter internalization incidence of the conidia after 4 h of incubation when compared to the wild-type (Figure 2B). However, the Δ*pksP* strain showed decreased internalization, whereas the complementation strain showed a similar internalization as the wild-type strain (Figure 2B). Similar to the wild-type, internalization of conidia of the Δ*rodA* and *pksP* complementation strains were reduced when the EphA2 receptor was inhibited by the EphA2 antibody (i.e., from 72 to 50% and 68 to 34%, respectively). In contrast, no inhibition was observed in the case of the Δ*pksP* strain (39 and 36% with and without inhibition, respectively). Inhibition of the EphA2 receptor with dasatinib did not reduce the internalization of the Δ*pksP* strain (26 and 22% with and without inhibition, respectively) but a decrease from 84 to 42% and 79 to 29% for the *pksP* complementation strain and the Δ*rodA* strain was observed, respectively (Supplementary Figure 6B). These results show that EphA2-dependent internalization of *A. fumigatus* conidia requires the presence of DHN-melanin.

### Dual Inhibition of Both EphA2 and Dectin-1

**FIGURE 3:**
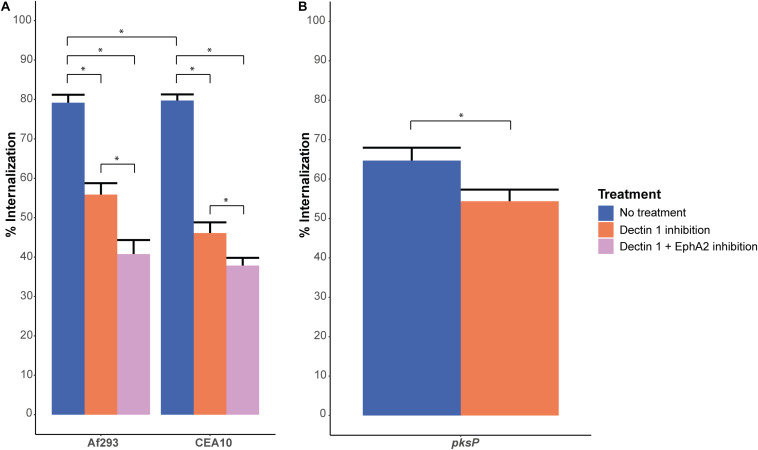
Internalization of conidia after EphA2 inhibition by dasatinib or Dectin-1 inhibition by the 2a11 antibody. **(A)** Internalization of *A. fumigatus* dormant conidia of strains Af293 (expressing *RFP*) and CEA10 WT (stained with *Aspergillus*-FITC antibody), after 4 h of infection without (blue bar) or, Dectin-1 receptor inhibition with 2a11 antibody (orange bar) or inhibition of both EphA2 (with an antibody) and Dectin-1 receptor (with 2a11 antibody) (pink bar). **(B)** Internalization of *A. fumigatus* CEA10 Δ*pksP* dormant conidia (stained with *Aspergillus*-FITC antibody), after 4 h of infection without (light bar) or with Dectin-1 receptor inhibition with 2a11 antibody (light gray bar). Bars represent the average of three separate experiments consisting of 10 pictures per condition/strain with the standard error. *Significant difference.

Dectin-1 is a β-glucan receptor like EphA2 and also plays a role in the internalization of *A. fumigatus* conidia into lung epithelial cells (Han et al., 2011). Inhibition of the Dectin-1 receptor with the 2a11 antibody resulted in a similar reduction of internalized conidia of Af293 and CEA10 WT as compared to EphA2 inhibition (Figure 3A); from 79 to 56% for Af293 and from 78 to 46% for CEA10 WT. Interestingly, inhibition of both Dectin-1 and EphA2 decreased internalization of conidia further to 41 and 39% for Af293 and CEA10, respectively (Figure 3A). Since the inhibition of the EphA2 receptor did not result in a decrease in internalization of the Δ*pksP* strain, we investigated if the internalization of conidia lacking DHN-melanin was also independent of Dectin-1. Inhibition of the Dectin-1 receptor with 2a11 antibody did result in a further decrease in internalization of conidia lacking DHN-melanin (Figure 3B). This decrease was less pronounced as observed with the wild-type strain, suggesting that the Dectin-1 receptor plays a less important role in the internalization of conidia lacking DHN-melanin.

## Discussion

**FIGURE 4:**
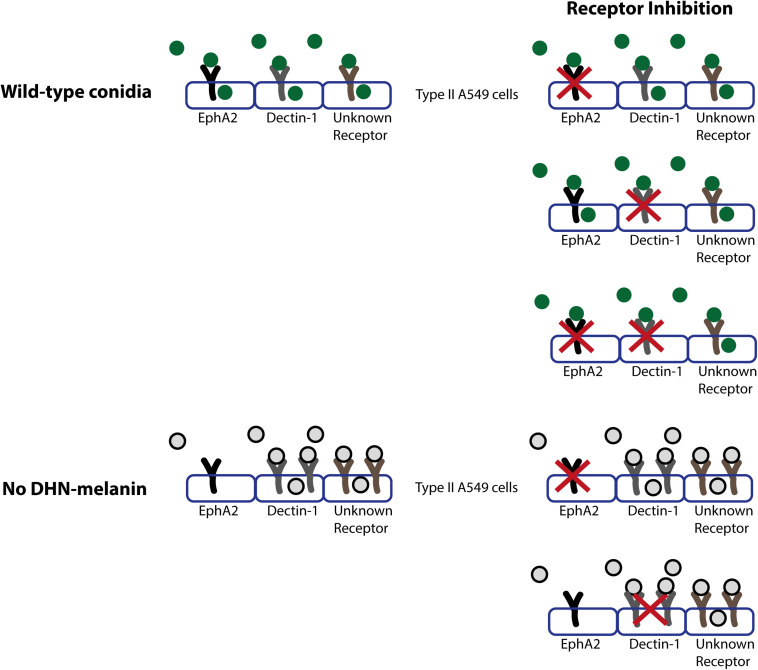
Model of the role of receptors in internalization and association of wild-type conidia (in dark green) or conidia lacking DHN-melanin (gray with black line). Conidia will associate to various indicated receptors present on the type II A549 epithelial lung cells, and the EphA2, Dectin-1 and at least one other receptor will be activated, which results in the internalization of conidia. In conidia lacking DHN-melanin, association of conidia is increased, and internalization of conidia is facilitated in an EphA2 independent and a Dectin-1 dependent manner.

The results of this study show that the β-glucan recognizing EphA2 receptor plays a role in the early interaction between *A. fumigatus* conidia and A549 epithelial cells next to Dectin-1 (Figure 4). Inhibition of the EphA2 receptor with the small molecule dasatinib leads to a 50% reduction in internalization. Since dasatinib not only inhibits the activation of the EphA2 receptor but acts as a broad range tyrosine kinase inhibitor, also inhibiting BRC-Abl and Scr and Syk family kinases (Schade et al., 2008; Baell and Holloway, 2010; Song et al., 2010), we also used an EphA2-specific antibody. Antibody-mediated inhibition gave similar results when compared to dasatinib. These results show that the EphA2 receptor is indeed involved in the internalization of *A. fumigatus* conidia, which is in line with the role of the EphA2 receptor in *C. albicans* (Swidergall et al., 2018).

The Syk family kinase is both inhibited via dasatinib and Dectin-1 inhibition. Interestingly, Syk signaling is also important for the maturation of phagolysosomes and fungal killing in monocytes (Kyrmizi et al., 2013), but a role in the internalization of conidia into epithelial cells has not been reported. It might be that part of the internalization of the conidia via the Dectin-1 receptor and the receptors inhibited by dasatinib is regulated via Syk signaling.

Differences in the association and internalization of conidia of the Af293 and CEA10 wild-type strains were observed in the A549 cell culture system. The conidia of Af293 have a lower association to the A549 cells and are more efficiently internalized when compared to CEA10 conidia (Figure 1). These differences are not due to the fact Af293 was expressing a red fluorescent plasmid and the CEA10 strain was labeled with an anti-*Aspergillus*-FITC antibody since labeling of the Af293.1 strain with this antibody did not alter internalization and association of conidia (results not shown). Differences between these two strains have also been reported in other infection systems. For instance, they differ in gene expression during an infection of airway epithelial cells (Watkins et al., 2018). Moreover, Af293 has a lower fitness at low-oxygen concentrations, which leads to a decrease in virulence in a mouse invasive pulmonary aspergillosis (IPA) model (Kowalski et al., 2016). The Af293 strain is also more virulent in a zebrafish model when compared to the CEA10 strain, which was explained by faster germination of CEA10 conidia and, consequently, a more rapid clearance by the host immune system (Rosowski et al., 2018).

The reduction of internalization of Af293 and CEA10 wild-type conidia after EphA2 receptor inhibition indicates an important role for β-glucan in the internalization of *A. fumigatus* conidia (Figures 1, 4). This internalization depends on the presence of DHN-melanin since internalization of a Δ*pksP* mutant was not affected by inhibition of EphA2 (Figures 2B, 4). It could be that conidia lacking DHN-melanin use alternative receptors, which remain to be identified. In this context, it is remarkable that inhibition of the Dectin-1 receptor results in a modest decrease in internalization of conidia lacking DHN-melanin. This suggests that Dectin-1 is mainly used as receptor for internalization if DHN-melanin is present (Figures 3B, 4). Notably, association of conidia lacking DHN-melanin with A549 cells was actually higher as compared to its wild-type and the complemented strain. This finding may be explained by changes in the cell wall architecture that impact interactions with cell surface receptors. How the presence of DHN-melanin affects the EphA2-dependent uptake remains to be clarified but it may be that a co-receptor for DHN-melanin interacts with EphA2. Absence of DHN-melanin could result in a shift to other receptors for binding and uptake. We observed that only 40% of the Δ*pksP* conidia were internalized indicating that a large amount of conidia remains cell-surface associated. Some alternative conidial surface components of *A. fumigatus* have been implicated in binding and internalization such as sialic acid (Warwas et al., 2007) and the CalA protein (Liu et al., 2017). Whether these surface components are involved in increased binding and/or internalization of conidia lacking DHN-melanin needs to be determined.

Dormant conidia expose small amounts of β-glucan at their surface. This amount is apparently sufficient for interactions with EphA2 because we did not find a relation between increased exposure of β-glucan and association and internalization. Swollen conidia of Af293 expose more β-glucan yet internalize with similar efficiency as compared to dormant conidia. Dormant conidia of strains lacking RodA also expose more β-glucan (Valsecchi et al., 2019) (Supplementary Figure 5), yet association and internalization was also similar as compared to WT conidia. Association of conidia lacking DHN-melanin is much higher as compared to WT and its complemented strain, while conidia of a Δ*pksP* strain expose much more chitin and glycoproteins at their surface, but not more ß-glucan (Valsecchi et al., 2019) (Supplementary Figure 5).

The increase in association of heat-killed Af293 and CEA10 conidia as compared to dormant conidia was reduced by inhibition of the EphA2 receptor. This difference in association might be due to differences in conidial surface structures after heat inactivation. On the other hand, no difference in internalization was observed between dormant and heat-killed spores, which is in line with the result of Wasylnka and Moore (2002). It should be noted that the internalization efficiencies of conidia into the epithelial cells differ between studies. This difference can be due to the use of different MOI’s, different *A. fumigatus* strains and/or time of exposure to epithelial cells. In our experiment, conidia which did not associate to the A549 epithelial cells were removed after 2 h of infection, meaning that only the associated conidia were used to determine the internalization. When the number of internalized conidia is determined based on the MOI at the start of the infection (Supplementary Table 1), it becomes clear that the internalization of the conidia is still higher than in other studies (Wasylnka and Moore, 2002).

In a macrophage model, it was observed that swollen conidia were more efficiently internalized when compared to dormant conidia, which was explained by the increased exposure of β-glucan (Luther et al., 2007). The reported 50% increase in internalization was not observed in our experiments (Figure 1B). This could be due to the difference in infection model and/or in swelling time and therefore the amount of exposed β-glucan. The swelling time for the macrophage model was 6 h, while we used 2 h in our experiments. Nevertheless, the 2 h swollen conidia in our experiments did show increased exposure of β-1-3 glucan (Supplementary Figure 5).

Since both EphA2 and the Dectin-1 receptor recognize β-glucan, we hypothesized that dual inhibition of both receptors completely abolishes internalization of conidia. This was not the case. It might be that other cellular receptors and processes sustain internalization under these conditions as is depicted in a schematic model in Figure 4. It is known that also actin dynamics in host cells is important for the internalization of conidia (Escobar et al., 2016; Culibrk et al., 2019). PLD activity is associated with actin dynamics and inhibition of PLD leads to a 50% reduction in internalization of conidia. Inhibition of the Dectin-1 receptor, leading to a reduction in internalized conidia, also leads to a reduction in PLD activity (Han et al., 2011). Further research in the involvement of actin and PLD activity in the internalization of conidia is required. *A. fumigatus* also has PLD isoforms. A PLD deletion strain of *A. fumigatus* has decreased intracellular and extracellular PLD activity and internalization of conidia is decreased, similar to PLD inhibition on A549 epithelial cells (Li et al., 2012). Aspf2 is another component of *A. fumigatus*, which could sustain internalization after EphA2 and Dectin-1 receptor inhibition. Aspf2 suppresses the host immune response by binding to plasminogen and to negative regulators of the complement system. The binding to plasminogen leads to increased damage of A549 cells after adhesion of the conidia. Deletion of Aspf2 leads to a decrease in A549 cell damage, but also to a decrease in internalization in macrophages (Dasari et al., 2018).

Interesting for future research is the MelLec receptor. This is, just as Dectin-1, a c-type lectin receptor, but instead of β-glucan it recognizes DHN-melanin (Stappers et al., 2018). The internalization of conidia lacking DHN-melanin is decreased and the observed internalization is partly via the Dectin-1 receptor but not the EphA2 receptor. More research is required to determine if the MelLec receptor is involved in the internalization of conidia and whether the decrease in internalization of conidia lacking DHN-melanin is due to the MelLec receptor unable to recognize these conidia.

The role of the EphA2 receptor in the transport of *C. neoformans* over the blood–brain barrier (Aaron et al., 2018) and the importance of EphA2 activation for the antifungal activity of neutrophils (Swidergall et al., 2019) highlights the important role of EhpA2 in fungal infections. It also questions the ability of this receptor to function as a druggable target for fungal infections. Inhibition of the EphA2 receptor may lead to a decrease in fungal uptake and crossing of the fungi over the blood–brain barrier but could also inhibit a proper neutrophil response against the fungal infection, therefore making it more difficult to control and clear the infection. To explore the potential of the EhpA2 receptor as a druggable target for fungal infections research should be done in more expanded models using immune cells following the processes that differ in these models (immune cell recruitment, fungal uptake, fungal proliferation, and killing).

Previously, an EphA2-dependent upregulation of IL-8 was observed with *C. albicans* (Swidergall et al., 2018). We therefore investigated whether an EphA2-dependent immune response of A549 cells could also be observed with *A. fumigatus*. We investigated IL-8 production after a 12 h co-incubation by an IL-8 specific ELISA. We did not observe an increased IL-8 production, which could support the proposed downregulating effect of *A. fumigatus* on the immune response by Escobar et al. (2018) (Supplementary Figure 7A). This contrasts other studies that did show induction of IL-8 (Oosthuizen et al., 2011; Chen et al., 2015). This might be explained by the fact that we remove non-associated conidia after 2 h of infection. Externally germinating conidia could be responsible for the induction of IL-8 in other studies. A decrease in IL-8 production upon EphA2 receptor inhibition as described by Swidergall et al. (2018) was not observed in our system after a 12 h inhibition with an EphA2 specific antibody (Supplementary Figures 7A,8A). Additionally, we investigated if A549 cells were damaged due to outgrowth of *A. fumigatus*. In line with the results of Seidel et al. (2020), we did not see an increase in cell damage expressed by LDH activity (Supplementary Figures 7B,8B), even though hyphae escaping A549 cells were observed (Escobar et al., 2018). This suggests non-lytic exit as described recently (Seidel et al., 2020). We cannot rule out the possibility that in our experimental setting the inhibition with the antibody did not last for the 12 h infection period. Alternatively, or in addition, other receptors might be involved to support the IL-8 production during inhibition of the EphA2 receptor.

Since the absence of DHN-melanin results in loss of EphA2-dependent uptake, it is tempting to speculate that sequentially interactions take place during germination at the lung epithelial surface. Dormant conidia containing DHN-melanin might be processed preferentially via the EphA2 receptor and internalized. Upon swelling and initiation of germination, DHN-melanin is gradually lost allowing a shift to other receptors. This shift might affect internalization, since absence of DHN-melanin results in increased surface association. This suggests that fast swelling and germinating conidia can be more efficiently cleared by the immune system than slow swelling and germinating conidia that seem to be taken up more efficiently by epithelial cells, thereby hiding from the immune system.

## Conclusion

It was shown previously that inhibition of Dectin-1 reduces internalization of conidia of *A. fumigatus* into epithelial cells. Here we showed that the EphA2 receptor is also involved in internalization of *A. fumigatus* conidia during infection. Next to EphA2 and Dectin1 also other receptors play a role in internalization since the dual inhibition of these β-glucan recognizing receptors did not lead to a complete block in internalization of conidia. In addition, we showed that DHN-melanin modulates association and internalization into lung epithelial cells. Its removal leads to a 60% reduction of internalization and becomes EphA2 independent while association to A549 cells is increased fivefold. Our results suggest a shift in receptor usage during swelling and germination at the lung cell surface affecting association and internalization.

## Data Availability Statement

All datasets generated for this study are included in the article/Supplementary Material.

## Author Contributions

EK and HC contributed to the design of the work. EK performed the experiments and wrote the manuscript. HW and HC made revisions. All authors were involved in the analysis and interpretation of data and approved the version to be published.

## Conflict of Interest

The authors declare that the research was conducted in the absence of any commercial or financial relationships that could be construed as a potential conflict of interest.

## Footnotes

1 www.fiji.sc

## Supplementary Material

Supplementary Figure 1 PCR confirmation of *pksP* complementation. Complementation transformant 4 was both positive in PCR for the *pksP* gene **(A)** and the presence of the reporter plasmid **(B)** and was used for all experiments.

Supplementary Figure 2 CFU counts after heat treatment (90°C, 20 min) of conidia. Bars represent the average of three separate experiments.

Supplementary Figure 3 Internalization and association of *A. fumigatus* conidia of strains Af293 (expressing the *RFP* gene) and CEA10 WT (stained with the *Aspergillus*-FITC antibody) after 4 h of infection, with (red bar) or without (blue bar) inhibition of the EphA2 receptor by the dasatinib. **(A)** Association of dormant, heat killed, or swollen Af293 and CEA10 conidia. **(B)** Internalization of dormant, heat killed, or swollen Af293 and CEA10 conidia. Bars represent the average of three separate experiments consisting of 10 pictures per condition/strain with the standard error.

Supplementary Figure 4 Internalization and association of *A. niger* conidia, expressing the *dTomato* plasmid. **(A)** Association of *A. niger* conidia after 4 h of infection, with (red bar) or without (blue bar) inhibition of the EphA2 receptor by dasatinib. **(B)** Internalization of dormant, heat killed, or swollen conidia after 4 h of infection, with (red bar) or without (blue bar) inhibition of the EphA2 receptor by dasatinib. Bars represent the average of three separate experiments consisting of 10 pictures per condition/strain with the standard error.

Supplementary Figure 5 β-Glucan exposure of dormant, swollen, and heat killed conidia of the Af293 and CEA10 wild-type strains and conidia of the *pksP* deletion and complementation strains and the *rodA* deletion mutant in the CEA10 background.

Supplementary Figure 6 Association **(A)** and internalization **(B)** of conidia of the CEA10 WT strain, the Δ*pksP* (lacking DHN-melanin) and Δ*rodA* (lacking the hydrophobin RodA) deletion strains, and the complemented Δ*pksP* strain after 4 h of infection with (red bar) or without (blue bar) inhibition of the EphA2 receptor by dasatinib. All conidia are stained with the *Aspergillus*-FITC antibody. Bars represent the average of three separate experiments consisting of 10 pictures per condition/strain with the standard error.

Supplementary Figure 7 IL-8 production (pg mL^–1^) of the A549 cells after 4 and 12 h of infections with *A. fumigatus* of CEA10, the Δ*pksP* (lacking DHN-melanin) and Δ*rodA* (lacking the hydrophobin RodA) deletion strains, and the complemented Δ*pksP* **(A)**. LDH activity of A549 cells after 4 and 12 h of infections with of CEA10, the Δ*pksP* (lacking DHN-melanin) and Δ*rodA* (lacking the hydrophobin RodA) deletion strains, and the complemented Δ*pksP* **(B)**. Blue bars represent cells which did not receive any treatment and EphA2 inhibition is shown by the red bars. Bars represent the average value of three independent experiments consisting of biological triplicates and technical duplicates.

Supplementary Figure 8 IL-8 production (pg mL^–1^) of the A549 cells after 4 and 12 h of infections with WT *A. fumigatus* conidia of Af293 and CEA10 at a MOI of 1 **(A)**. LDH activity of A549 cells after 4 and 12 h of infections with WT *A. fumigatus* conidia of Af293 and CEA10 at a MOI of 1 **(B)**. Blue bars represent cells which did not receive any treatment, EphA2 inhibition is shown by the red bars, Dectin-1 inhibition by the orange bar, and dual inhibition of the EphA2 and Dectin-1 receptor is represented by the pink bar. Bars represent the average value of three independent experiments consisting of biological triplicates and technical duplicates.

Supplementary Table 1 Conidia removed by washing. Numbers are averages of three independent experiments, with 10 pictures in each experiment and at least 100 counted conidia. The association and internalization data is also used for the graph in Supplementary Figure 1.

## Abbreviations

BSA: bovine serum albumin
DHN: dihydroxynaphthalene
DMEM: Dulbecco’s Modified Eagle Medium
EGFR: epidermal growth factor receptor
EphA2: ephrin type-A receptor 2
FBS: fetal bovine serum
LDH: lactate dehydrogenase
MOI: multiplicity of infection
OM: osmotic medium
PDA: potato dextrose agar
PLD: phospholipase D
ROS: reactive oxygen species.
