# Supplementary material for: EphA2-Dependent Internalization of A. fumigatus Conidia in A549 Lung Cells Is Modulated by DHN-Melanin
Source: Front Microbiol. 2020 Oct 6;11:534118. doi: 10.3389/fmicb.2020.534118 (PMC7573251; doi:10.3389/fmicb.2020.534118)
Supplement: Supplementary Figure 1 — PCR confirmation of pksP complementation. Complementation transformant 4 was both positive in PCR for the pksP gene (A) and the presence of the reporter plasmid (B) and was used for all experiments. [file Data_Sheet_1.docx]

**Supplementary Table 1**

|  | Af293 | CEA10 |
| --- | --- | --- |
| Start (conidia/cell) | 1 | 1 |
| Association after 4 hours (including washing) (conidia/cell) | 0.08 | 0.25 |
| Removed by washing (conidia/cell) | 0.92 | 0.75 |
| Internalized conidia (%) of the associated conidia | 81 % | 75 % |
| Internalized conidia (conidia/cell) of the associated conidia | 0.0648 | 0.1875 |
| Internalized conidia (%) of the start inoculum | 6.48% | 18.75% |


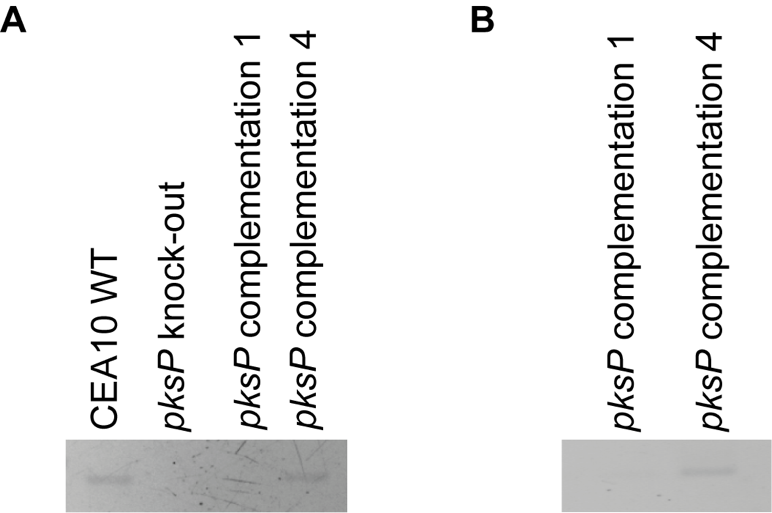


**Supplementary Figure 1**


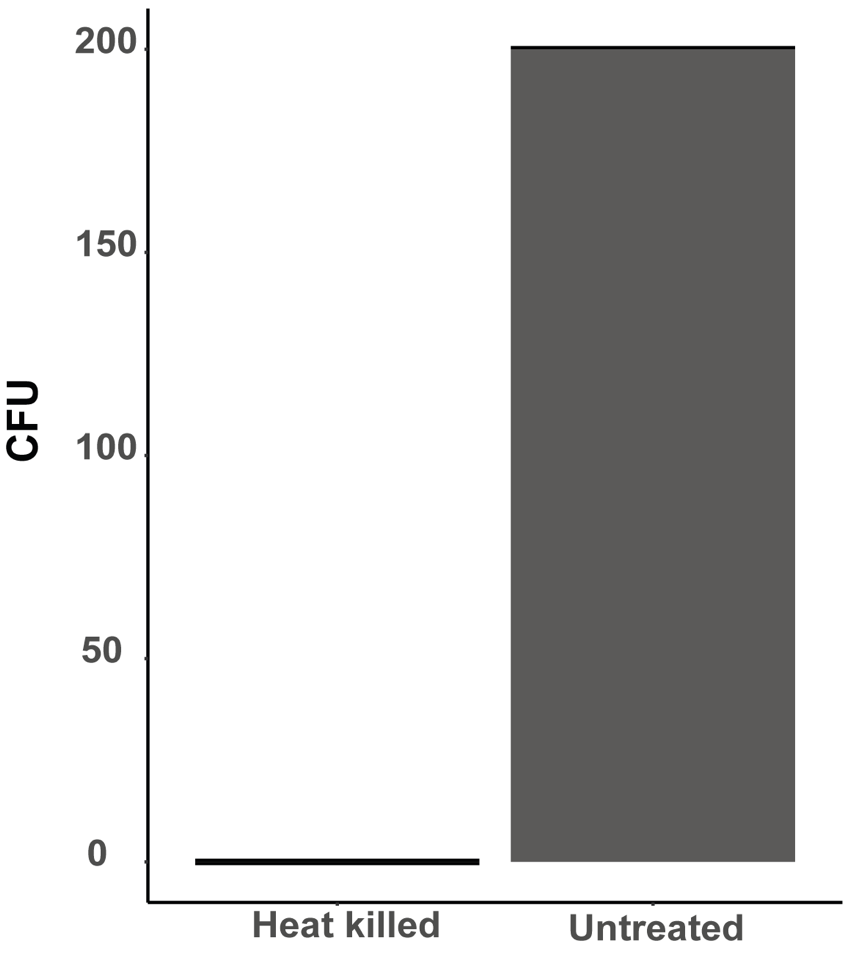


**Supplementary Figure 2**


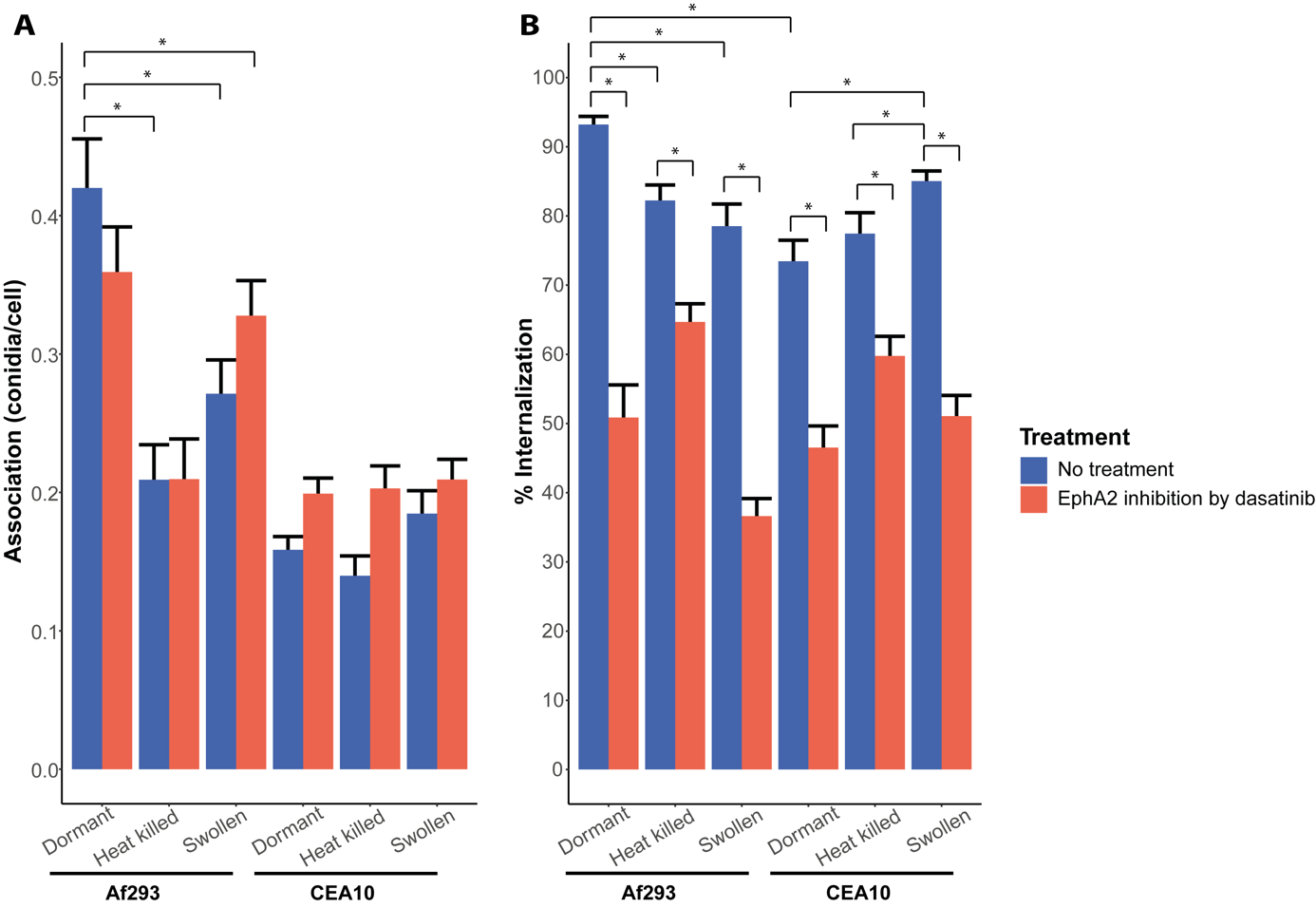


**Supplementary Figure 3**

**
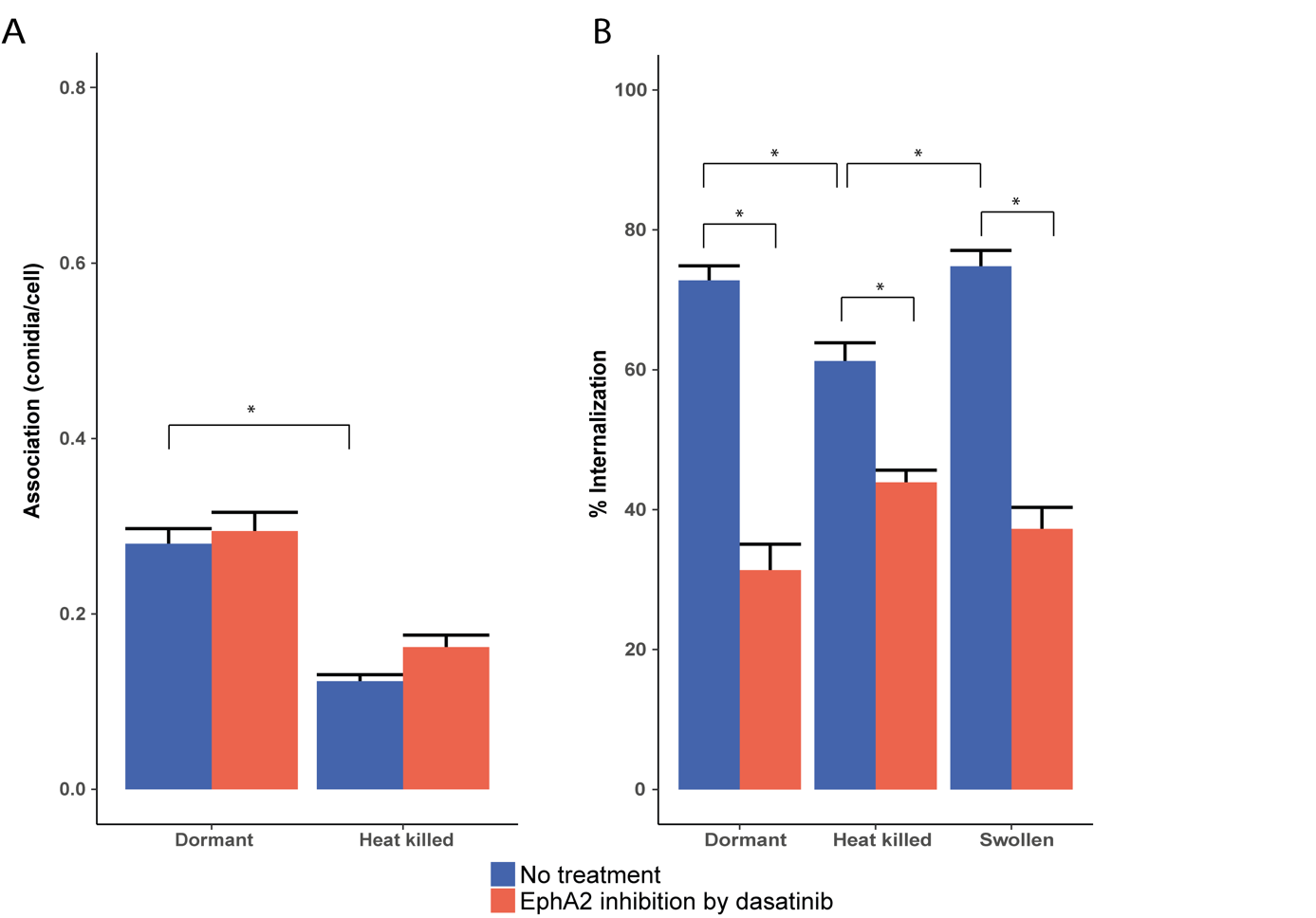
**

**Supplementary Figure 4**


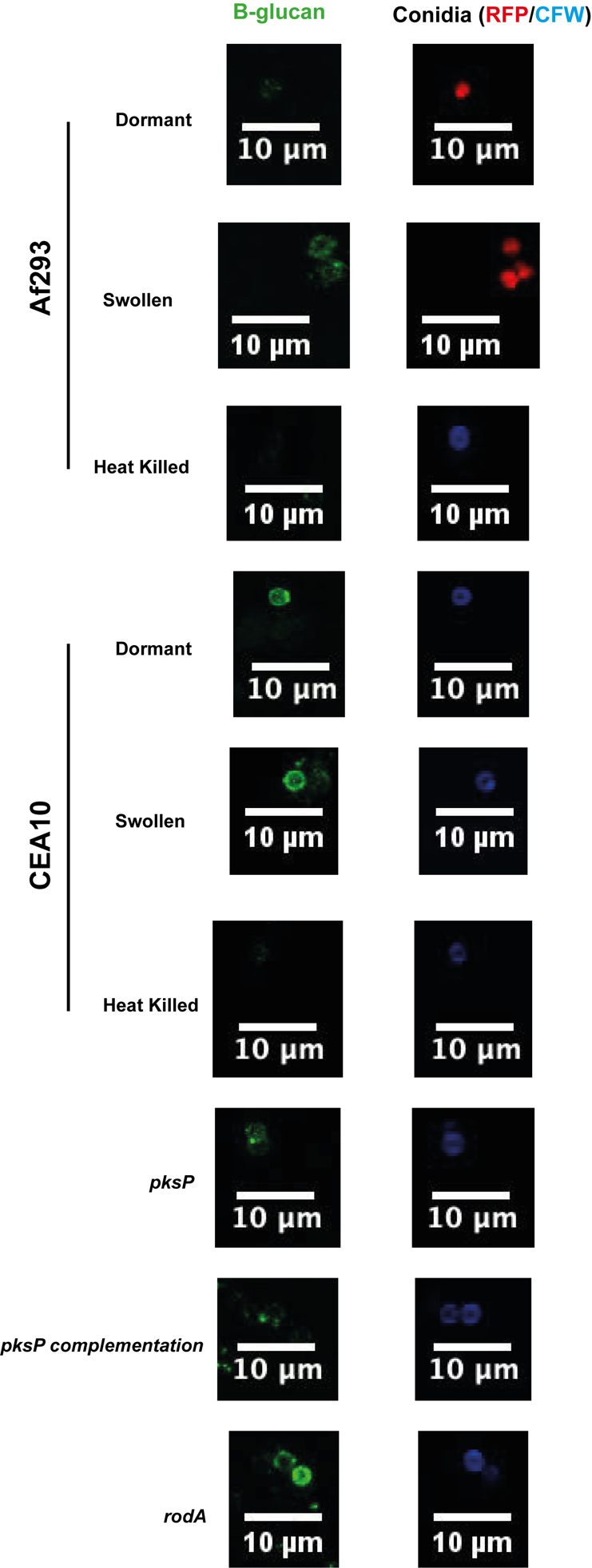


**Supplementary Figure 5**


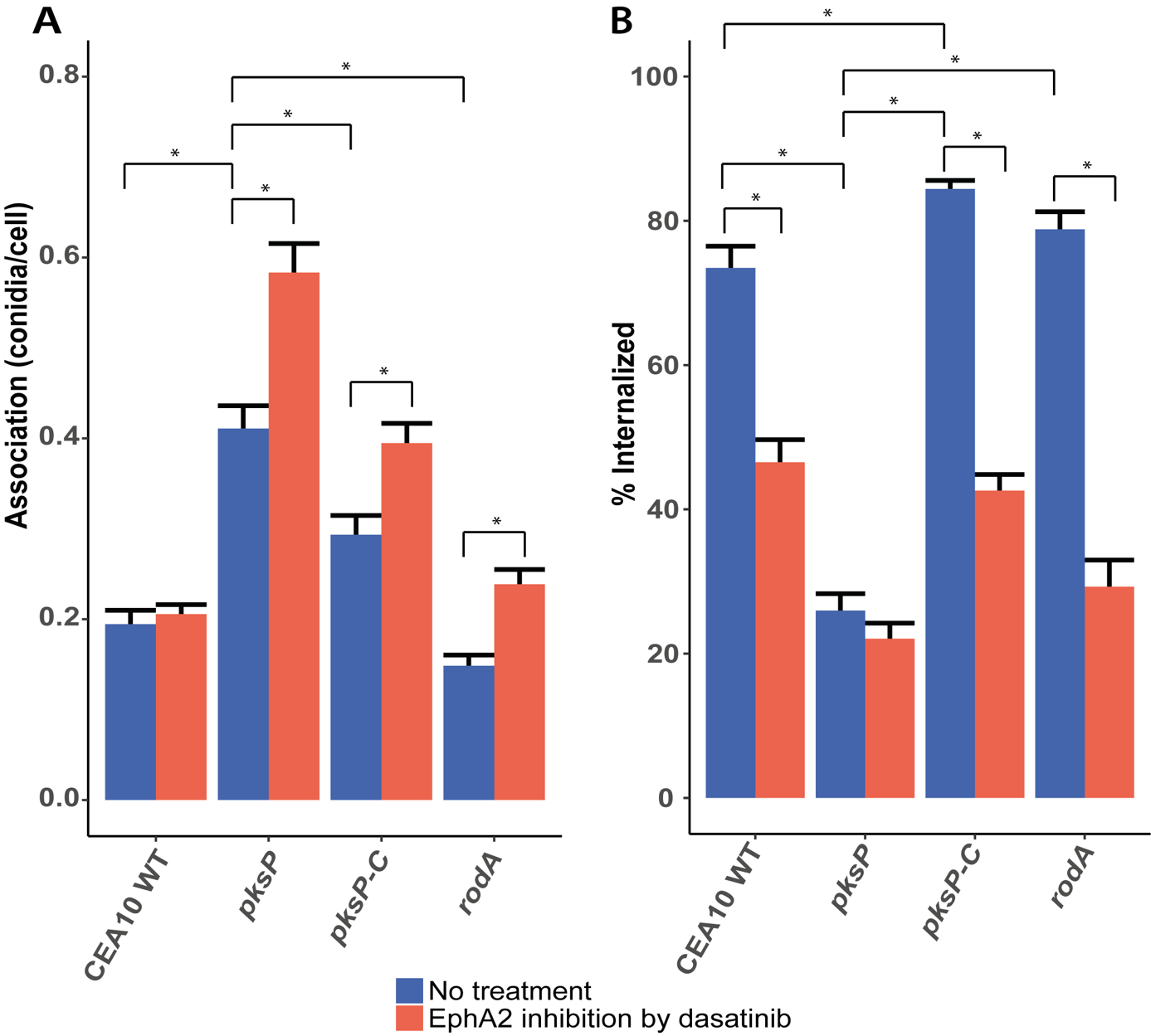


**Supplementary Figure 6**


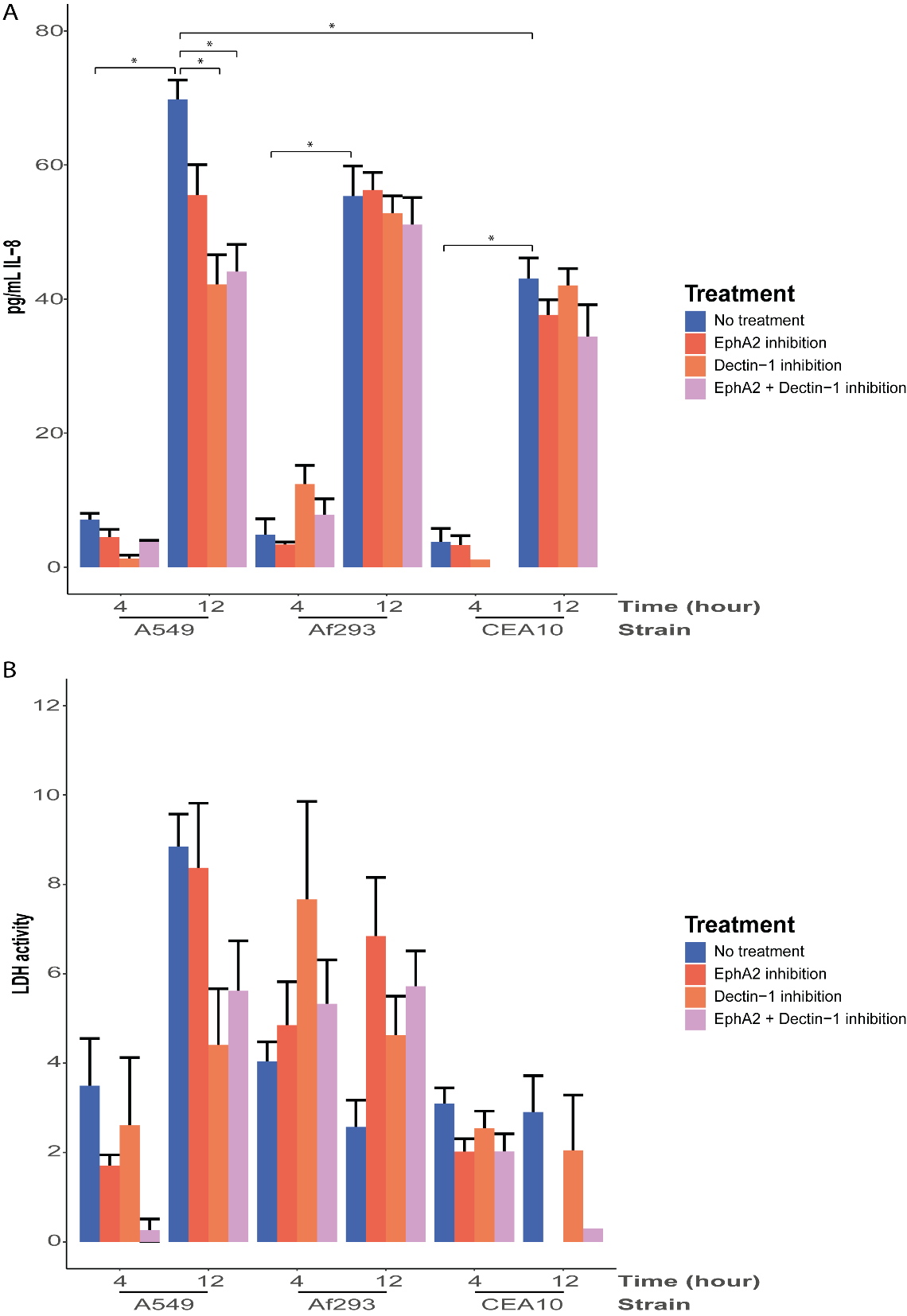


**Supplementary Figure 7**


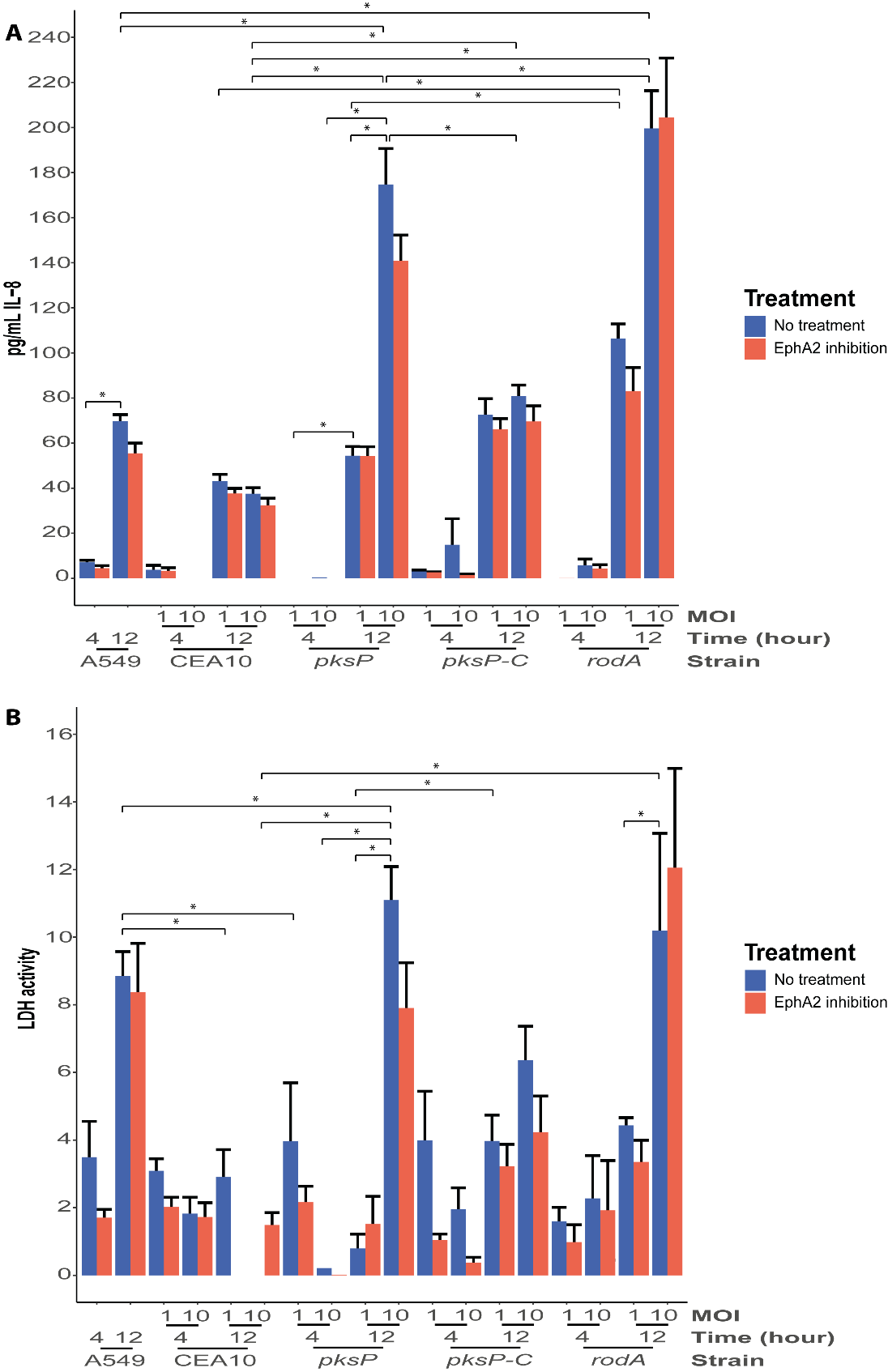


**Supplementary Figure 8**
